# Current role of coronary calcium in younger population and future prospects with photon counting technology

**DOI:** 10.1093/ehjci/jeac214

**Published:** 2022-11-17

**Authors:** Filippo Cademartiri, Pàl Maurovich-Horvat

**Affiliations:** Department of Radiology, Fondazione Monasterio/CNR, via Giuseppe Moruzzi, 1 - 56124, Pisa (PI), Italy; Department of Radiology, Medical Imaging Centre, Semmelweis University, Budapest, Üllői út 26, 1085Hungary

**This editorial refers to ‘Age-stratified effects of coronary artery calcification on cardiovascular and non-cardiovascular mortality in Korean adults’, by J. Kang *et al*., https://doi.org/10.1093/ehjci/jeac184.**

Cardiac computed tomography (CCT) is progressively becoming a cornerstone of cardiovascular medicine not only because of its prominent role in chronic coronary syndrome (CCS) but also because of it robustness in the field of structural heart disease treatment planning, of revascularization strategy planning, and, last but not least, of primary prevention. Coronary atherosclerosis/coronary artery disease (CAD) has been shown more and more as continuum between normality and severe obstructive disease, with several different qualitative and quantitative features along the spectrum.^1–3^

In this issue of the Journal, Kang *et al*.^4^ deliver a very extensive study on the predictive value of CACS in younger adults. The authors investigated both cardiovascular and non-cardiovascular mortality in 160 821 individuals in a quite young population (mean age = 41.4 years; 73.2% young individuals aged <45 years) in primary prevention;^4^ the median follow-up was 5.6 years. This unique and very robust set of data grants us an insight into something we are not used to look in this way. CACS has mostly been regarded as a tool for cardiovascular risk stratification to be used later in life, when usually, in developed countries, everyone starts to check a bit more upon his/her actual health. Epidemiologically, we already know CAD is important in early stages of life and it is affected by our habits. What is progressively changing is the attitude towards cardiovascular disease; we are collectively becoming more aware of the fact that CAD is curable and eventually preventable; we are starting to see drugs that can stop the progression of CAD and its complications and hence we want to start early to prevent greater damage in a later stage of life.

The authors of the study show us that we probably should investigate a more personalized/individualized strategy for cardiovascular disease prevention at an earlier stage in life, especially if we consider the potential role of CACS; in this cohort below the age of 45 years, the weight of coronary calcification on prognosis is very high.

Some limitations need to be highlighted: (i) the lack of analysis beyond mortality (cardiovascular events) which may significantly add to the comprehension of the distribution of CAD and its complications vs. age; (ii) the lack of further analysis of the impact of gender; we know gender is heavily affecting the impact of CAD through decades of life, and this is reflected in the distribution and amount of CACS; (iii) individuals were aware of the results of the CACS, and this may have affected subsequent behaviours and hence prognosis; and (iv) ethnic specificity for young Koreans who can afford a primary prevention programme.

The literature about the role of CACS and the relationship between CACS and biomarkers, other plaque components and radiomics have been studied for some time.^5–11^ Several additional information has been produced but still much more investigation have to be performed.

**Figure 1:**
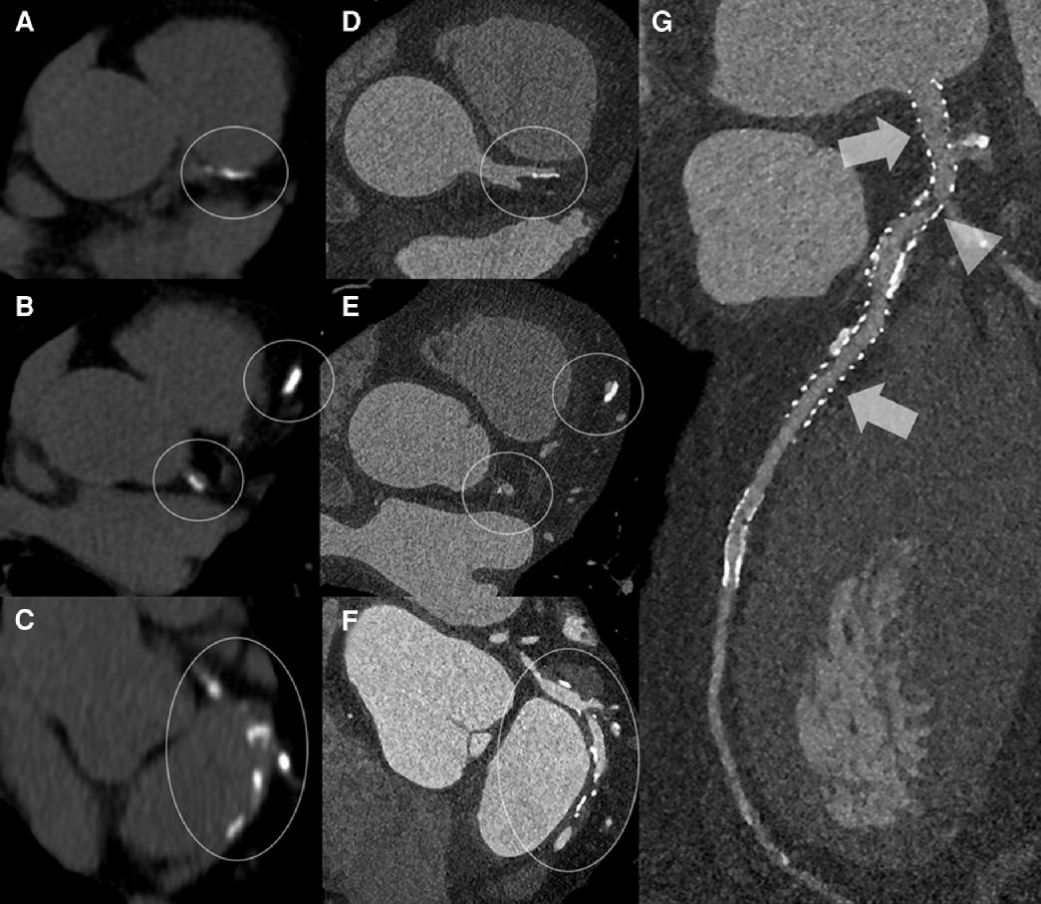
The figure shows examples of CAD as displayed by conventional calcium scoring protocol (*A*, *B*, *C*; inside white circles and ellipses) and corresponding CT coronary angiography images (*D*, *E*, *F*; inside white circles and ellipses) obtained with photon counting CT—PCCT technology (NAEOTOM ALPHA, Siemens Healthineers). It is very clear that the detection and phenotyping of calcifications is going to be dramatically improved by this technology. These improvements will also determine a much better visualization of high attenuation/density structures such as coronary artery stents (*G*) in which even stent structs will be easily differentiated (arrows) together with in-stent re-stenosis (arrow-head).

As already stated above, CT technology has progressed and continues to progress, and we are on the verge of the introduction at the clinical level of a detector technology (i.e. photon counting detector) that will impact significantly the detection, characterization, and quantification of coronary calcifications.^12–14^ This technology will change the way we look at calcifications by allowing detection of smaller calcifications (spatial resolution 100–200 µm) and being able to better quantify the amount calcium anywhere in the coronary tree and off course within and around the cardiac chambers, within cardiac valves, and so forth (*Figure 1*). This change will probably affect the measurement methods, and probably current CACS methodology will be updated.

In conclusion, CACS is an important clinical parameter for risk stratification in young individuals; it must be studied more and larger cohorts. Newer technologies will bring more insight into the significance of CACS in different contexts.
